# The Recent Development in Chemoresistive-Based Heterostructure Gas Sensor Technology, Their Future Opportunities and Challenges: A Review

**DOI:** 10.3390/membranes12060555

**Published:** 2022-05-26

**Authors:** Mir Waqas Alam, Pheiroijam Pooja, Muhammad Aamir, Basma Souayeh, Shehla Mushtaq, Muhammad Shuaib Khan, Muhammad Nasir Amin, Kaffayatullah Khan, Shanavas Shajahan

**Affiliations:** 1 Department of Physics, College of Science, King Faisal University, Al-Ahsa 31982, Saudi Arabia; bsouayeh@kfu.edu.sa; 2 Department of Electronics and Communication Engineering, National Institute of Technology Nagaland, Chumukedima, Dimapur 797103, India; pheiroiipj93@gmail.com; 3 Department of Basic Science, Preparatory Year Deanship, King Faisal University, Al-Ahsa 31982, Saudi Arabia; msadiq@kfu.edu.sa; 4 School of Natural Sciences, National University of Sciences and Technology, Islamabad 44000, Pakistan; shehla.mushtaq@sns.nust.edu.pk; 5 International Research Center for Renewable Energy (IRCRE), State Key Laboratory of Multiphase Flow in Power Engineering (MPFE), Xi’an Jiaotong University, 28 West Xianning Road, Xi’an 710049, China; m.shuaibkhan@mail.xjtu.edu.cn; 6 Department of Civil and Environmental Engineering, College of Engineering, King Faisal University, Al-Ahsa 31982, Saudi Arabia; mgadir@kfu.edu.sa (M.N.A.); kkhan@kfu.edu.sa (K.K.); 7 Department of Chemistry, Khalifa University, Abu Dhabi P.O. Box 127788, United Arab Emirates; shanavas.shajahan@ku.ac.ae

**Keywords:** gas sensors, semiconductors, polymers, nanomaterials, metal oxides

## Abstract

Atmospheric pollution has become a critical problem for modern society; therefore, the research in this area continually aims to develop a high-performance gas sensor for health care and environmental safety. Researchers have made a significant contribution in this field by developing highly sensitive sensor-based novel selective materials. The aim of this article is to review recent developments and progress in the selective and sensitive detection of environmentally toxic gases. Different classifications of gas sensor devices are discussed based on their structure, the materials used, and their properties. The mechanisms of the sensing devices, identified by measuring the change in physical property using adsorption/desorption processes as well as chemical reactions on the gas-sensitive material surface, are also discussed. Additionally, the article presents a comprehensive review of the different morphologies and dimensions of mixed heterostructure, multilayered heterostructure, composite, core-shell, hollow heterostructure, and decorated heterostructure, which tune the gas-sensing properties towards hazardous gases. The article investigates in detail the growth and interface properties, concentrating on the material configurations that could be employed to prepare nanomaterials for commercial gas-sensing devices.

## 1. Introduction

With the increase in demand for energy and economic growth, there is continuous emission of several pollutants, namely, H_2_S, NO_x_, NH_3_, CH_4_, SO_x_, CO, and fluorocarbons, from industries and automobile exhaust into the environment, creating many health problems [1,2]. To minimize and control the harm resulting from air pollution, there is a need to monitor the units that detect these factors and determine pollutants within the range of standard norms. In 2018, at the Global Conference on Air Pollution and Health, the General Director of the World Health Organization, Dr. Tedros Adhanom Ghebreyesus, stated that air pollution is a “silent public health emergency” and “the new tobacco” [3]. Therefore, detecting and observing flammable and toxic pollutant gases are required to safeguard human health and protect the environment [4,5]. Throughout the years, several types of gas sensors have emerged that employ numerous sensing materials with diverse transduction platforms [6,7,8]. In the 1960s, Seyama tried to demonstrate that gas sensing can be done with ZnO thin-film electrical devices [9]. He employed chemoresistive ZnO thin films working at a temperature of 485 °C. The chemoresistive thin films showed approximately a 100-times detection response to propane in comparison to the thermal conductivity gas sensor. In 1970, Taguchi synthesized the first practical application of a chemoresistive gas sensor with tin dioxide (SnO_2_) [10]. With the pioneering work conducted by Seyama and Taguchi, many researchers have started working with and proposing the use of gas-sensing devices with varied functionalities. Metal oxides are regarded as the material of choice for a gas sensor owing to their low cost, high stability, ease of fabrication, and high sensitivity [11,12]. With the advancement in nanotechnology, researchers have focused on low-dimensional nanoparticles and quasi-one-dimensional (1D) structures, namely, nanowires and nanotubes [13]. Nanostructured metal oxide semiconductors enhance the signal sensing due to their large surface area and because they are stable at a high operating temperature in comparison to their bulk form. The issue with metal oxide semiconductor sensors is the baseline resistance drift during the interactions, which lessens the surface accessibility to reactive gases, resulting in low selectivity [14]. This difficulty may be partially resolved by combining an array of different sensors, also called an “electronic nose”, to simultaneously analyze several signals at once [15]. Combining various metal oxides or using them with different materials, such as graphene, conducting polymers, etc., to form heterostructures has been found to improve the selectivity and exhibit unique gas-sensing characteristics [16,17,18,19,20,21,22,23,24]. Different fabrication methods can be employed to synthesize one-dimensional (1D) and two-dimensional (2D) layered heterojunctions, core–shell nanostructures, and three-dimensional (3D) hierarchical structures [25,26,27,28,29,30,31]. 

Despite advancements in nanotechnology and material science and the outcomes achieved with heterostructure gas sensors, further investigations and studies are needed, such as those focusing on the reproducibility of the fabrication process, possible diffusion across the junction, synergistic effect among the material forming the heterostructure, and stability of the contact. Consequently, a careful investigation of the growth and the interfacial properties of gas-sensing nanomaterials can fill the present gap in the laboratory studies and the real-world exploitation of heterostructures. This review aims to provide insights on the growth and use of different structures for gas-sensing nanomaterials that detect specific types of gas operating at room temperature or at high temperatures, where it can be applied commercially.

## 2. Performance of Gas Sensors

The performance of gas sensors has been analyzed using different parameters, such as response time, selectivity, sensitivity, recovery time, stability, and fabrication cost [32,33]. Selectivity is the capability of a gas sensor to recognize a specific gas present in the gas mixture. Sensitivity is a change in the measured signal per analyte concentration unit, i.e., the slope of a calibration graph—that is, the sensor response versus the target gas concentration. Response time, T_res_, gives the duration when the concentration of the gas reaches 90% of the total response of the signal, such as resistance upon exposure to the target gas. Reversibility refers to the ability to check whether a gas detector returns to its starting state after the gas returns to its normal concentration level [33]. Recovery time, T_rec_, is the time it takes for a gas sensor to return to 10% of the final value after the removal of the measured variable. It represents the ability of a gas sensor to reproduce the output for a certain period of time. These results include retaining selectivity, sensitivity, and response along with recovery duration. Ideally, a gas detector should show high sensitivity, stability, fast response, fast recovery, high selectivity, and low cost of fabrication [32]. 

## 3. Gas-Sensing Devices

**Figure 1:**
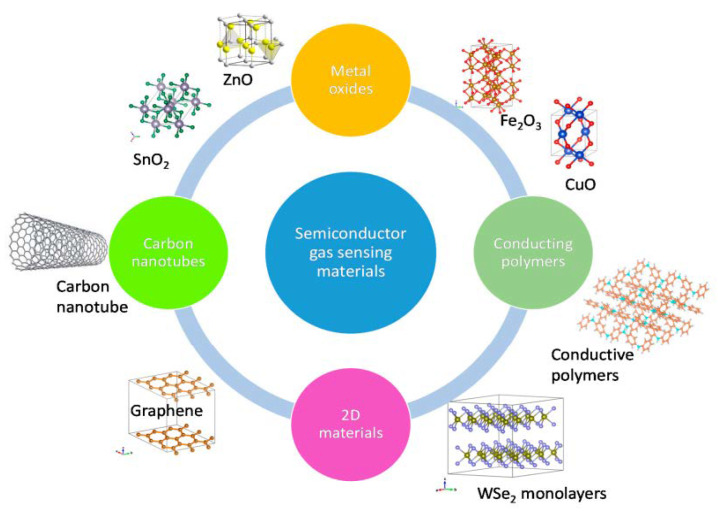
Semiconductor gas-sensing materials. Reprinted with permission from Ref. [35]. Copyright 2020, Nikolic, M.V. et al.

Over the past years, several gas sensors have been designed based on different sensing materials and different methods. Gas sensors are classified as electrochemical, catalytic combustion, infrared absorption, thermal conductive, solid electrolyte, paramagnetic, and metal oxide semiconductor sensors [34], depending on the change in electrical resistance with different materials and their compositions. Semiconducting metal oxide, polymers, carbon nanotubes (CNTs), graphene, etc., are mostly used (Figure 1). 

Gas sensors can also be classified based on optic, acoustic, calorimetric, and chromatography methods. According to Comini [36], a gas sensor can be classified based on the measurement methods, such as (1) DC conductometric gas sensors, (2) photoluminescence (PL)-based gas sensors, and (3) field effect transistor (FET)-based gas sensors. Table 1 illustrates the comparison of various gas sensors studied by Korotcenkov [37]. Target gas detection by a metal oxide semiconductor has gained wide attention because of its various advantages over other types of sensors. Electrochemical gas sensors have become unpopular due to their short lifespan, limiting their use to only a few applications. Although an optical gas sensor has good sensitivity, an adequate lifespan, good selectivity, and fast response, the cost is high and the size is large. Although metal oxide semiconductor gas sensors are less selective, the low cost and simplicity in fabricating them are the main factors that contribute to their wide usage. 

## 4. Gas-Sensing Materials

### 4.1. Metal Oxide Semiconductors

**Figure 2:**
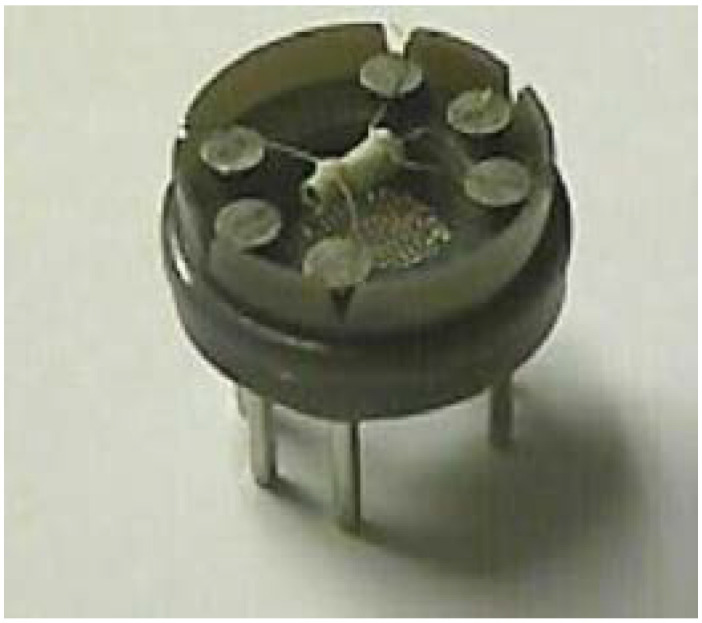
Taguchi chemoresistive gas sensor. Reprinted with permission from Ref. [38]. Copyright 2015, Neri, G.

A metal oxide semiconductor gas sensor is constructed using a sensitive material; it can also be fabricated on a suitable support, where the gas molecules are recognizable. The gas molecule can be recognized either on the surface of the sensing material or present in the material. The change in concentration of the sensing element can be transformed into an electrical signal. This simple sensing mechanism process enables the device to be fabricated using several configurations. The Taguchi gas sensor uses a sintered porous sensitive ceramic body material (Figure 2). 

**Figure 3:**
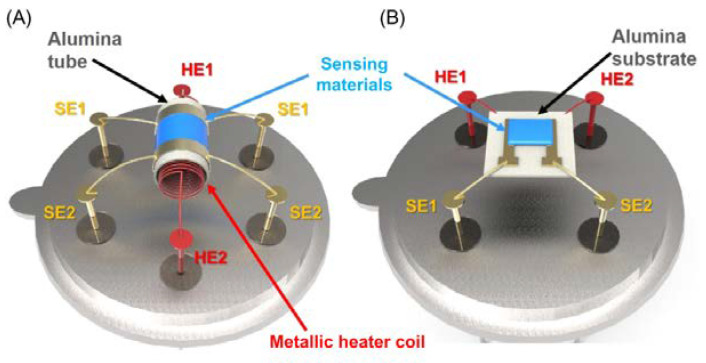
Schematic structure of (**A**) tube-type and (**B**) substrate-type oxide semiconductor gas sensors (SE, sensor electrode; HE, heater electrode). Reprinted with permission from Ref. [39]. Copyright 2019, Lee, J.-H.

Other examples of metal oxide semiconductor gas sensors with different forms and dimensions are displayed in Figure 3. The slurry of the gas-sensing materials can be deposited onto an alumina ceramic tube and then sintered to an elevated temperature in order to form a layer of thick sensing film. The temperature is monitored using a metal-coil heater present within the tube, as shown in Figure 3A. The planar form of gas sensors consists of a thick/thin layer of sensing material deposited onto a ceramic substrate using physical or chemical methods employing interdigited electrodes, as shown in Figure 3B.

**Figure 4:**
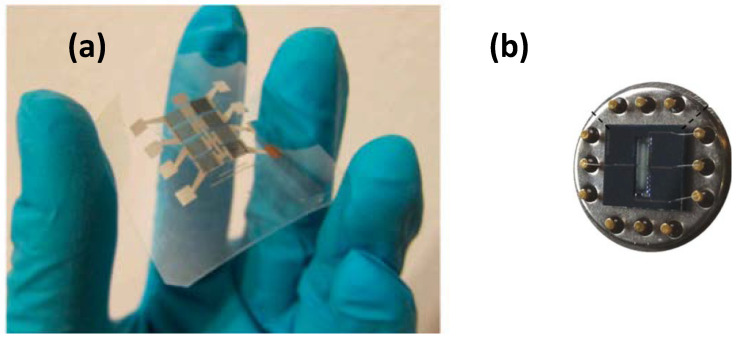
(**a**) Flexible gas sensor. Reprinted with permission from Ref. [40]. Copyright 2014, Lorwongtragool, P. and (**b**) micromachined gas sensor. Reprinted with permission from Ref. [41]. Copyright 2014, Stoycheva, T. et al.

The sensing material layer is synthesized onto a plastic support, thus enabling the formation of flexible metal oxide semiconductor gas sensors, as shown in Figure 4a. These gas detectors are small; however, there is an increased demand for miniaturized gas sensors for various applications, particularly to make them compatibile with integrated circuit (IC) technology. IC devices are synthesized with a top-down approach, which results in higher throughput for large-scale integration. Using this process, micromachined metal oxide semiconductor gas sensors can be designed on a chip substrate, allowing easy interface with the conventional Si micro-electronics (Figure 4b). The development of finer microstructures has led to the creation of a synthesized nanosize gas sensor [37]. 

Metal oxide-based gas sensors sense gas by monitoring the variations in electrical signals due to gas. The model of the adsorption or desorption process is the most-followed gas-detecting mechanism, focusing on chemical or physical adsorption and desorption of the analyte gas on the sensing surface, causing a change in the material resistance attributed to the variation in the concentration of the charge carrier. When metal oxides are exposed to air, the oxygen (O_2_) molecules get adsorbed on the material surface. When O_2_ is adsorbed on the metal oxide surface, the molecules tend to reside in the O_2_ vacancies formed previously and then take up electrons from the conduction band of the material to obtain negative O_2_ species.

O_2_ (g) → O_2_ (ad) (1)

O_2_ (ad) + e^−^ → O_2_^−^ (ad) (2)

O_2_^−^ (ad) + e^−^ → 2O^−^ (ad) (3)

O^−^ (ad) + e^−^ → O^2−^ (ad) (4)

**Figure 5:**
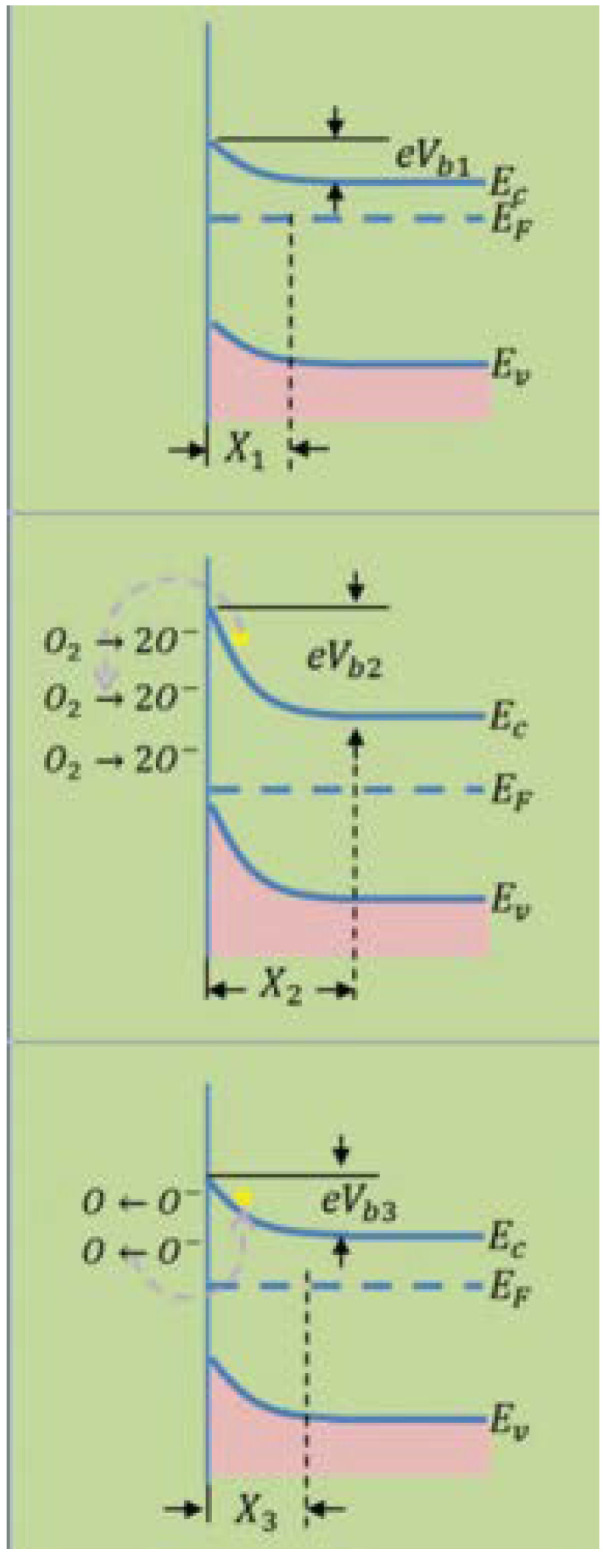
Schematic energy band diagram of SnO2 during the gas-sensing reaction. Reprinted with permission from Ref. [42]. Copyright 2016, Li, T. et al.

After consuming the electrons that are present in the conduction band, a depletion layer of electrons is formed. The O_2_ adsorption causes the Fermi level to slide downward and bend the conduction band upward. Here, with the arrival of the target gas, it experiences a redox reaction with the negative O_2_ species liberating electrons again into the conduction band. There is shrinkage of the depletion layer, the O_2_ vacancies re-form, the conduction band and the Fermi level to return to normal, and resistivity is regained, as shown in Figure 5 [42]. In metal oxide semiconductors, the main charge carriers are the electrons, as in n-type materials, such as TiO_2_, ZnO, WO_3_, and particularly SnO_2_, or holes in p-type materials, such as CuO and NiO [43]. Most research has investigated n-type metal oxide semiconductors for chemical sensing [44]. However, p-type metal oxides are less often examined than n-type metal oxide semiconductors, due to the lower performances associated with the nature of charge carriers [45,46]. When the metal oxide surface is exposed to reducing elements, such as hydrogen, acetone, and ethanol, there is a decrease in n-type resistance, but p-type resistance increases. Due to interactions with oxidizing elements, such as ozone and carbon dioxide, electrical resistance increases in n-type semiconductors and decreases in p-typesemiconductors. The advancement of nanotechnology has enhanced the phenomena and knowledge of existing materials at the nanoscale. In the early 2000s, the research community prioritized metal oxide gas sensors on zero-dimensional nanoparticles and 1D structures, namely, nanowires, nanorods, and nanobelts [13]. 

Nanostructured metal oxide semiconductors enhanced the sensing of the signals due to their large surface-like nature and their stability at high working temperatures.

Solution synthesis techniques are known to be simple yet powerful ways to prepare semiconductor nanomaterials. Hydrothermal synthesis is considered to be the most researched technique, enabling the synthesis of a variety of metal oxides [47]. This is carried out by chemical reactions and variations in the solubility of the substances present in aqueous solution under ambient pressure and temperature. This synthesis is done in an autoclave in which the concentration of the solution, the time, and the temperature of the reaction are controlled [48]. Solvothermal [49], pulse laser deposition (PLD) [50], atomic layer deposition (ALD) [51], RF sputtering [52], thermal evaporation [53], E-beam evaporation [54], electrospinning [55], the facile solution method [56], sol-gel [57], and chemical vapor deposition (CVD) [58] are also employed for the synthesis of metal oxide nanostructure films.

**Figure 6:**
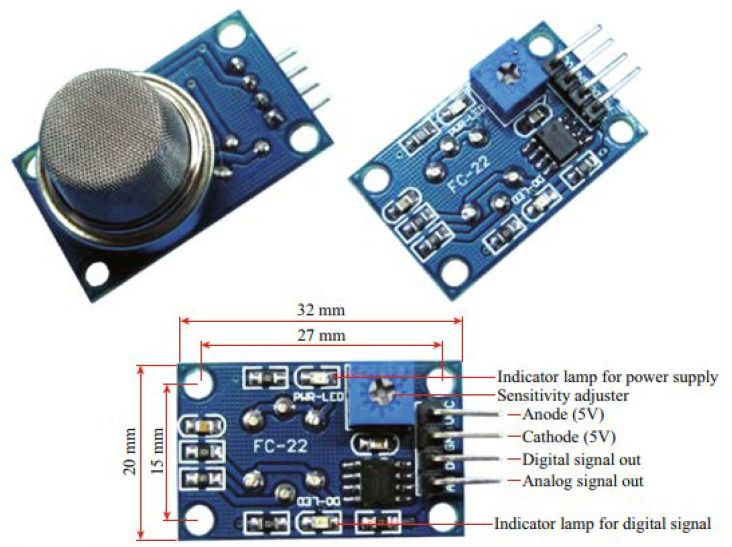
SnO_2_ gas sensor for detecting CO; model: MQ-9.

SnO_2_ is a widely researched n-type metal oxide material that can be applied in many practical commercial devices. It is a wide band gap semiconductor (3.6 eV) known for its good electrical charateristics [59]. It is highly sensitive to different gas species and it enables the detection of low-concentration gaseous species. Figure 6 displays an MQ-9 module of an SnO_2_ gas sensor for detecting CO, which is readily available on the market. However, it suffers from a lack of selectivity. A titania (TiO_2_) n-type semiconductor is known for its gas sensing due to its lesser cross-sensitivity to humidity in comparison to various metal oxides [60]. TiO_2_ has been explored as a resistive oxygen gas-sensing layer working at temperatures ranging from medium to high for air or fuel automotive ratio control. At 400–600 °C temperatures, the detection of oxygen is largely attributed to the reaction occuring on the surface, whereas for temperatures between 700 and 1000 °C, the detection of oxygen is mostly attributed to oxygen ions diffused in the bulk materials [61].

Copper oxide (CuO), a p-type semiconductor where the holes are most of the carriers, has a high response to gas that cannot be obtained in n-type semiconductors. Here, the key parameters for obtaining high sensitivity to gas is the high interparticle connection [62]. The p-type semiconductor metal oxides, CuO, NiO, Mn_3_O_4_, and Cr_2_O_3_, catalyze the oxidation of volatile organic material, thus attaining excellent selectivity to those gases [35]. An additional benefit of a p-type gas sensor is its humidity tolerance. The p-type semiconductor nickel oxide (NiO) (3.6–4.2 eV) material withstands and works at temperatures above 500 °C and can sense H_2_ [63]. It also exhibits a high ratio response to NH_3_, a quick response to recovery in temperatures ranging between 250 and 350 °C for the thin film synthesized by employing RF sputtering [64]. NiO nanowires show good gas-sensing operation towards toluene, acetone, ethanol, methanol, and triethylamine at 350 °C [65], whereas NiO nanowires synthesized by employing a catalyst, such as Pt and Au, exhibited improved sensing to H_2_ and CO at 300 °C and at 500 °C for acetone [66]. The p-type chromium oxide (Cr_2_O_3_) (3.4 eV) has been explored for use as a gas sensor [67], where enhanced sensing of toluene is obtained by the Cr_2_O_3_ microspheres [68].

### 4.2. Conducting Polymers

In 1983, the properties of conducting polymer sensors were first reported. The gas sensor employed doped polypyrrole (PPy) operating as an ammonia sensor [69]. Now, conducting polymers, poly (3,4-thylenedioxythiophene), polyaniline (Pani), and polythiophene (PTh), with their composites, are employed in chemical sensors due to their advantageous properties, such as versatility, low cost, high sensitivity, low working temperature upon exposure to various gases, and ease in processing variations in electrical and optical properties [70,71,72,73,74,75]. Conducting polymer sensors convert the analyte into observable signals: resistance, absorption, waves, or audible variables. When the sensor is exposed to the analyte vapor, the interaction occurs between the gas molecules and the polymer sensor material, which causes variations in the physical properties of the sensor’s material. Electron acceptors, such as NO_2_, have the ability to detach electrons from the aromatic conducting polymer rings. During this event, the electric conductance and the level of doping in the p-type polymers (PANI or PPy) increases. Consequently, their electrical resistance decreases. This process can be elaborated upon with the support of the following equation.

NO_2_ (gas) + e^−^ → NO_2_^−^ (ads) (5)

**Figure 7:**
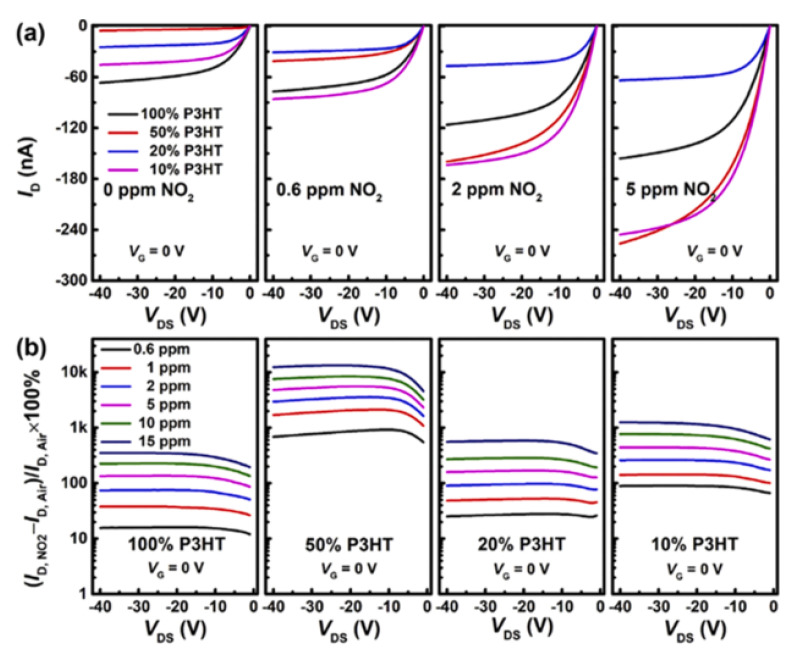
Variations of I_D_ and μ of the indicated P3HT mass fractions at the given NO_2_ concentrations with different carrier gases. Reprinted with permission from Ref. [76]. Copyright 2018, Yang, Z. et al.

Based on the equation given above, the concentration of electrons on the polyaniline surface are reduced. In fact, the conductivity of a pure conducting polymer is rather low. Thus, to achieve higher conductivity and make it suitable for sensing, a doping procedure is necessary. Poly (9-vinylcarbazole) (PVK) and poly(3-hexylthiophene-2,5-diyl) (P3HT) are the two semiconducting polymers widely used as an active layer in organic field effect transistors (OFETs) for the sensing of NO_2_, as depicted in Figure 7 [76]. 

Rakesh et al. studied the synthesis of polyaniline (PANI) film under a room-temperature condition for sensing NO_2_. With ammonium persulphate employed as the oxidant and aniline as the monomer, using the chemical oxidative polymerization process, the PANI film was successfully synthesized and it exhibited excellent sensitivity of 12.10% at 100 ppm of NO_2_, showing a rapid response of ~11 s and a recovery of 7 min, respectively [77]. Matindoust et al. investigated the gas-sensing characteristic of PANI films, where the films were coated using polyimide on a flexible sheet. PANI films were studied at several humidities and variations in the NH_3_ gas flow ranging from 50 to 150 ppm to sense the odor emitted from meat spoilage. The gas sensor displayed high sensitivity and a quick NH_3_-sensing response [78]. Liu et al. studied a PANI nanofiber–metal oxide gas detector for detecting CO in hydrogen. In that study, the gas-sensing device was fabricated using an interdigitated electrode. It showed a good sensing response to a 1 ppm concentration of CO in hydrogen under 25 °C [79]. Karmakar et al. investigated a NO_2_ gas sensor employing a silver–polypyrrole nanocomposite (Ag-PPy). The Ag-PPy thin film doped using 15% PTSA (p-Toluene sulfonic acid) exhibited more selectivity and sensitivity to NO_2_ at room temperature. It displayed a good sensing response of 68% at 100 ppm NO_2_, showing a 148 s response time and a 500 s recovery time [80].

### 4.3. Graphene and Carbon Nanotubes

Since 2004, scientists have shown great interest in manufacturing graphene, its modifications, and their applications in several fields [81], due to its outstanding characteristics, such as single-atom thick 2D conjugated structures, stability at room temperature, large specific surface areas, and ballistic transport. High conductivity and ballistic transport enabled graphene to exhibit less signal hindrance when operated as a gas sensor [82]. It does not necessitate electric heating, due to its chemically stable nature under room temperature for chemical sensing [83,84] All these features enable graphene to be a potential candidate for gas sensing. There are four major ways to fabricate single-layer and multilayered graphene: micromechanical exfoliation, vapor deposition, epitaxial growth, and chemical reduction [85,86,87]. In 2007, the first experiment using graphene for sensing gas molecules was approved. Schedin et al. discovered that a micrometer-sized graphene gas sensor can detect single gas molecule adsorbed to or detached from the graphene’s surface [88].

**Figure 8:**
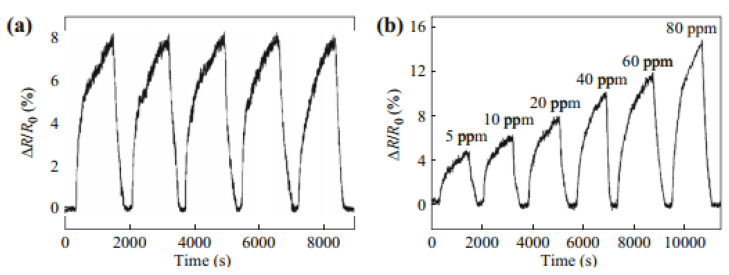
(**a**) Reproducibility of CRG sensors to 20 ppm DMMP vapor. (**b**) The response curve of the CRG sensor to dimethyl methylphosphonate (DMMP) vapor under a 5–80 ppm concentration range. Reprinted with permission from Ref. [91]. Copyright 2012, Hu, N. et al.

Choi et al. reported on the use of multilayered graphene to effectively sense NO_2._ The multilayered graphene films were fabricated employing the CVD method using a flexible substrate embedded with a microheater [89]. It exhibited a low NO_2_ detection limit at 200 ppb levels and a quick response time upon exposure to 1 ppm NO_2_ at room temperature. Nemade et al. developed a graphene-based CO_2_ sensor [90]. They synthesized layered graphene employing an electrochemical exfoliation method that displayed high stability, quick response, fast recovery times, and a low detection limit. This layered graphene also displayed excellent sensing characteristics to liquid petroleum gases. Figure 8 hows the real-time response of dimethyl methylphosphonate (DMMP) vapor detected by a para-phenylene diamine reduced graphene oxide-based vapor sensor. Its excellent repeatability, superior selectivity, and low limit of detection are displayed in Figure 8 [91].

Researchers have been employing CNT as a material for gas sensors for 20 years [92]. The seamless cylinders obtained using graphene sheets wrapped in the axial direction form a single-wall CNT (SWCNT) if all the atoms present in one sheet act as surface atoms. In a multi-wall CNT (MWCNT), many sheets are present and the atoms on the outermost layer account for the sensing response [93]. CNTs have a high surface area-to-volume ratio and good mechanical properties, chemical stability, and electronic properties that are reliable for gas-sensing applications. They are considered to be the next generation of gas sensors that can bring a change in the market [94]. CNTs are highly sensitive to the gases that donate or withdraw electrons, such as NH_3_ and NO_2_. However, there are difficulties in technologies that hamper their commercialization; the synthesis is expensive and it is challenging to grow defect-free nanotubes [95].

SWCNT can be produced using the CVD method [96] and the arc-discharge method [97]. Wongwiriyapan et al. developed a SWCNT network gas detector that was synthesized on Al_2_O_3_ substrates using a CVD setup [96]. Prior to the CVD process, a Fe (0.5 nm) thin-film layer was synthesized on an Al (6 nm) layer on the substrate using the electron-beam evaporation method. On the back of the substrate, a Pt layer was formed that worked as a heater. The SWCNT was deposited using CH_4_ (99.99%) as the carbon source at 53 kPa pressure and a 300 sccm flow rate at 850 °C for 5 min. The deposited SWCNT network had a uniform diameter covering the Al_2_O_3_ substrate region. The SWCNT network exhibited good sensitivity to NO_2_ gas at a concentration of 50 ppb.

**Figure 9:**
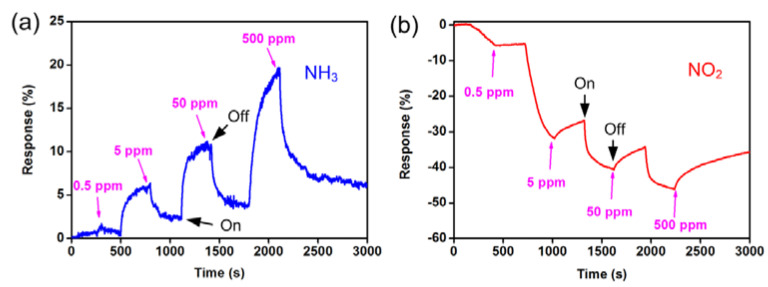
Performance of gas sensor devices based on the SWCNT networks grown with the nanoporous silica support layer. The sensor was sequentially exposed to 0.5 @500 ppm levels of (**a**) NH_3_ and (**b**) NO_2_ in nitrogen. Reprinted with permission from Ref. [99]. Copyright 2012, Han, Z.J. et al.

In 2000, Kong et al. found that there are three orders of magnitude changes in the electrical conductivity of semiconductors when they are put under NO_2_ and NH_3_ gases [92]. SWCNT exhibits a p-type semiconducting nature with a Fermi level at 25 meV above the valence band, resulting in a semiconducting performance at room temperature and resistances of 0.3–5 MΩ [98]. Due to the adsorption of NO_2_ molecules on the SWCNTs, there is a transfer of charge to NO_2_ from SWCNT, as NO_2_ molecules tend to seize more electrons. In the case of NH_3_, a lone pair of electrons is collected by the SWCNTs. Hence, the conductivity of SWCNTs rapidly increased when exposed to NO_2_ and decreased when exposed to NH_3_. The SWCNT network displayed good sensitivity to NH_3_ and NO_2_ under room temperature, as displayed in Figure 9a,b, respectively [99]. There was an increase in resistance as the NH_3_ concentrations increased. The sensors displayed quick response time and fast recovery time. The response to NO_2_ sensing is also shown in Figure 8b. Compared to NH_3_, there was a decrease in device resistance. The resistances were reduced by 7%, 32%, 41%, and 46% at various respective concentrations of 0.5, 5, 50, and 500 ppm for NO_2_.

MWCNTs were also explored in gas-sensing applications. Ahmadi et al. designed gas sensors using MWCNTs. MWCNTs were synthesized using the electrophoretic deposition (EPD) technique on a polytetrafluoroethylene (PTFE) membrane in non-aqueous media [100]. Furthermore, the MWCNT layer was formed using paste and employing doctor blade deposition. From the experimental results, it can be concluded that the EPD method required a lower amount of MWCNT in comparison to the doctor blade method. Valentini et al. employed plasma-enhanced CVD (PECVD) to synthesize MWCNT film on Si_3_N_4_/Si substrates and form NO_2_ gas sensors. The resistivity decreased when exposed to NO_2_ gas. The sensor worked at an optimal temperature of 165 °C and it detected a low sensing limit of 10 ppb. It showed quick response and good selectivity. Furthermore, MWCNT-based sensing behavior could be enhanced with heat treatment in oxygen [101]. The semiconducting nature of MWCNT film changed to metallic after heat treatment in oxygen. It was found that when exposing the device to NO_2_, the electrical resistance decreased suddenly. Chung et al. observed that the electrical resistance of an MWCNT with a large diameter decreased in air after electrical breakdown, whereas no sensitivity was seen if no electric breakdown occurred [102]. Hence, MWCNT sensitivity could be controlled by the degree of electrical breakdown. MWCNTs with a larger diameter showed higher sensitivity, because MWCNTs with a large diameter have more damage in the shells during an electrical breakdown, thus forming more places for oxygen molecule adsorption.

**Figure 10:**
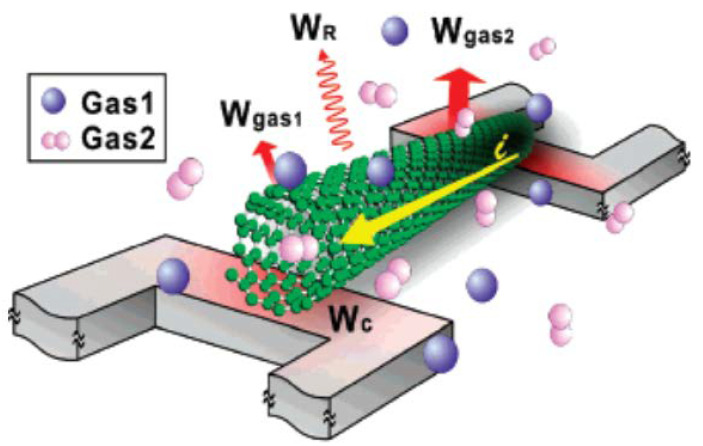
Schematic diagram displaying the architecture and gas-sensing mechanism of suspended CNT. Reprinted with permission from Ref. [103]. Copyright 2007, Kawano, T. et al.

Kawano et al. built a gas sensor using the electrothermal effect of MWCNTs placed within two Si microbridges as seen in Figure 10 [103]. Placing the Joule-heated MWCNT under different levels of gas pressure, the device resistance changed depending on the conductive heat transfer nature of the gas molecule. The electrothermal MWCNT gas sensor was sensitive to different concentrations of gas used and also helped in differentiating the gases. Moreover, the MWCNT gas sensor showed quick reversible responses, small volume, and low power consumption with the provision of integrating it with microelectronic fabrication processes. Figure 10 displays the schematic of the MWCNT electrothermal gas-sensing principle, where a single MWCNT is present between two Si microbridges. The system is heated using electrical current. The total heat generated was divided into three parts: heat conduction to the two microbridges (*W*C), heat radiation (*W*R), and heat transfer through the gases (*W*_gas1_ and *W*_gas2_ for two gases having different thermal conductivities). Since the heat-transfer process is associated with the gas pressure and the gas item, the change in temperature and thus the change in resistance of the MWCNT would be dependent on the gas pressure and gas item.

## 5. Strategies for Improving Gas-Sensing Properties

### 5.1. Bilayer or Multilayered Heterostructure

A heterostructure is basically a physical as well as an electronic junction present among two dissimilar materials. As materials connect, electrons, which are at a higher energy level, tend to flow along the interface to unoccupy the lower energy states until the Fermi levels are alligned. This results in the formation of a depleted charge-carrier region at the interface. A potential barrier builts at the junction owing to the band bending, due to the variation in the Fermi level of the material. After overcoming the potential energy barrier, the charge carrier moves through the interface. This combination of various materials provides a unique way to enhance the structural and functional properties, thus enhaning the structure’s gas-sensing capabilities. 

**Figure 11:**
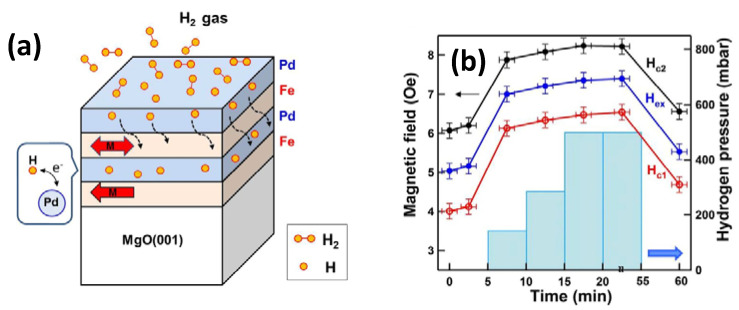
(**a**) Schematic diagram of the effect of hydrogenation on Pd-covered Fe/Pd/Fe trilayers on Mg(001) substrate. (**b**) Left: magnetic field; right: corresponding H_2_ gas pressure in the measurement environment. Reprinted with permission from Ref. [104]. Copyright 2018, Hsu, C.-C. et al.

A heterostructure on bilayer or multilayered films exhibits a clear interface, which is suitable for characterization and can be readily modeled due to the easy stacking of the 2D material. The thermal stability in these structures can be easily investigated and the possible growth of mixed phases can be studied due to the diffusion along the interface [90]. Hsu et al. studied [Pd/Fe]_2_ multilayers formed on MgO(001) to investigate the hydrogen impact on the magnetic interlayer coupling [104]. In this work, the complex magnetic hysteresis nature, consisting of single, double, and triple loops, was analyzed as a function of the azimuthal angle in a transverse direction and a longitudinal direction. With the exposure to hydrogen gas, the separation of two minor loops increased due to the creation of Pd-hydride, enhancing the ferromagnetic coupling among the two Fe layers. Upon applying a favorable constant magnetic feld, there is a reversible 90° rotation of the top-Fe moment following hydrogen exposure, suggesting that the Pd space layer employed for mediating the magnetic interlayer coupling is highly sensitive to hydrogen; consequently, this multilayered system can work as a giant magnetoresistance sensor applicable for hydrogen sensing, as depicted in Figure 11a,b.

Verma et al. synthesized a highly sensitive H_2_S gas sensor using a multilayered SnO_2_/CuO thin film and pulse laser deposition (PLD) [50]. In this work, sequential laser ablations were employed for each material for every 10 layers. The contents of the material were found by changing the laser pulses. There was enhanced H_2_S detection using SnO_2_ with 3 vol% of CuO content, forming SnO_2_/CuO multilayered films. The creation of *p*-*n* junctions as well as the adsorbed O_2_ species enabled higher electrical resistance of the multilayered SnO_2_/CuO thin-film devices in comparison to single SnO_2_ thin-film devices. Furthermore, a *p*-*n*-*p*-*n* multilayered interface was made, resulting in different depletion regions. CuO changed to CuS upon exposure to H_2_S. CuS possesses metallic properties, causing shrinkage of the space charge regions at the interfaces of CuO/SnO_2_. It was found that the reduced depletion width allowed more charge carriers to flow through the SnO_2_ layer. Maximum response was found with 3 vol% of CuO multilayered structures; this was attributed to the decrease in porosity of the film with the increase in the content of CuO.

Zhang et al. fabricated a high-performance sandwich CuO–rGO–CuO H_2_ sensor using the hydrothermal technique and drop-casting method [105]. Two sensor groups were prepared, one with three-layer (CuO–GO–CuO) and one with five-layer ((CuO–GO)_2_–CuO) structures. For comparison, sensors on pure GO along with CuO films were also designed. All the films were heat treated under 180 °C for 3 h in order to reduce GO into rGO. Of all the devices, the CuO–rGO–CuO film gas sensor displayed the highest performance to H_2_ over a wide concentration range. This is because the conductivity of bare CuO was lower and the capability of the hydrogen molecule captured by bare rGO was weaker in comparison to the CuO–rGO–CuO film. The (CuO–rGO)_2_–CuO film thickness was greater than the CuO–rGO–CuO hybrid film, so the electrons disappeared in the multi-layer transfer process, leading to a weaker performance in comparison to the CuO–rGO–CuO film.

Liang et al. reported on humidity sensing using sputtered ZnO and In_2_O_3_; their multilayered thin films were anlayzed by impedance measurement [106]. They found that the chemisorption of H_2_O molecules can tune the sensor impedance in low relative humidity conditions even with the high-impedance bare In_2_O_3_ and ZnO films. They also found that the increase in the concentration of relative humidity allowed Grotthuss chain reactions to occur on the surface of the films. The adsorbed H_2_O molecules led to free hydrogen ions hopping among the H_2_O molecules, releasing H_3_O^+^ by dissociating H_2_O. In the multilayered ZnO/In_2_O_3_ thin film, the existence of a heterojunction and enhanced charge density increased the humidity-sensing performance. 

Larin et al. reported on the comparison of an H_2_S sensor between bare, bilayer, and multilayered SnO_2_/TiO_2_ films [107]. It was found that the sensor response decreased when the content of TiO_2_ in the multilayered sensors increased due to the agglomeration of the TiO_2_ content into larger grains, which decreased their catalytic activity. 

Hoa et al. built a gas detector using hybrid graphene and NiO nanosheets. They reported two orders higher sensitivity in comparison to the bare NiO nanosheet devices for NO_2_ sensing at the 1 ppm level [108].

Pasha et al. worked on the synthesis of PEDOT-PS reduced graphene oxide organic thin flms for NH_3_ gas sensing at room temperature; 10% rGO-doped PEDOT-PSS thin films showed enhanced sensitivity with a rapid response and recovery duration of 1.05 and 2.84 min for different concentration of NH_3_ gas from 200–1000 ppm [109]. 

### 5.2. Mixed Compounds

Mixed compounds have received a lot of attention due to the ease in their preparation techniques. The compounds are obtained by combining two or more materials with the help of chemical or physical methods. Evans et al. reported on MWCNTs and SWCNTs modified using VO_2_ for sensing H_2_O vapor and NH_3_ at room temperature [110]. The VO_2_–SWCNTs and VO_2_–MWCNTs were synthesized by employing a continuous hydrothermal flow synthesis. First, the powder of SWCNTs or MWCNTs was spread in a salt solution of vanadium to synthesize the hybrids. The VO_2_-CNTs gas sensors showed excellent sensitivity in comparison to pure VO_2_. Both the composition of the surface as well as the microstructure had a significant effect on the performance of the hybrid gas detector. Zhou et al. described the synthesis of an rGO/Cu_2_O nanocomposite for an H_2_S gas sensor that can detect gas at a low limit of 5 ppb under room temperature. This was due to the large adsorption of H_2_S molecules on the surface and the absence of any capping surfactant [111]. Wang et al. reported on a room-temperature NH_3_ sensor using CeO_2_ nanoparticles@cross-linked PANI, which showed a fast response time and high long-term stability. The nanohybrids exhibited an enhanced sensing response under room temperature from NH_3_ in the range of 6.5 to 50 ppm, owing to the formation of *p*-*n* junctions through the interaction between both of these materials [112]. PANI is the most prominent material amongst the conducting polymers employed for highly selective and sensitive materials for low-concentration NH_3_ sensors at room temperature. Eising et al. employed PANI to synthesize a hybrid MWCNTs-PANI film for an NH_3_ gas sensor [113]. The hybrid was modified using three different dopants: camphorsulfonic acid, sulfuric acid, and *m*-cresol. This gas sensor exhibited higher sensing than the bare PANI device, which was attributed to the stability of MWCNTs. The MWCNTs-PANI film doped with camphorsulfonic acid showed the highest sensitivity. These hybrid sensors demonstrated an NH_3_ gas-sensing limit of 4 ppm at room temperature. 

**Figure 12:**
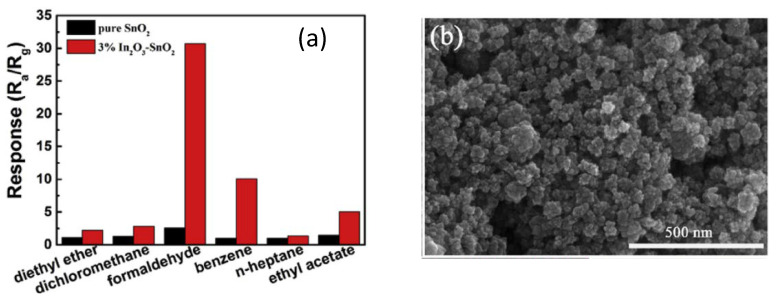
(**a**) Selectivity of pure SnO_2_ and 3% In_2_O_3_-SnO_2_ nanospheres towards 100 ppm of various VOC gases at 100 °C and (**b**) SEM image of 3% In_2_O_3_-SnO_2_ nanostructure. Reprinted with permission from Ref. [114]. Copyright 2018, Ge, W. et al.

Ge et al. investigated porous hybrid In_2_O_3_-SnO_2_-sensing nanostructures synthesized successfully using the facile hydrothermal technique and calcination [114]. The hybrid structures with 3% In_2_O_3_ showed a higher response of 30.7–100 ppm formaldehyde detection at 100 °C, 14 times greater than pure SnO_2_. The In_2_O_3_-SnO_2_ nanostructure gas sensor exhibited good stability and selectivity for formaldehyde. Here, the improved formaldehyde sensing characteristics were due to the n-n heterojunctions and the synergistic interaction between the In_2_O_3_ as well as SnO_2_ in the hybrid nanostructure, as depicted in Figure 12a,b.

Shaposhnik et al. reported on the comparison of hydrogen sensing using TiO_2_-doped SnO_2_ produced by coprecipitation and mechanical mixing, respectively; this yielded similar randomized dispersions [115]. The optimal composition for sensing H_2_ was 10 mol% of TiO_2_ for the coprecipitation and 20% of TiO_2_ mixing mechanically. Thus, composition has a significant impact on the gas-sensing performance. 

Bai et al. reported on the gas detection response by varying the content of WO_3_ in the mixed compound of WO_3_-SnO_2_ produced using the sol precipitation technique [116]. The compound obtained with 20% of WO_3_ showed a high NO_2_-sensing response value ranging from 186 to 200 ppm. However, the sensing response was reduced slowly as the WO_3_ content in the compound decreased or increased. Substitutional ion dissolution into the lattices of other metal oxides could be seen in the mixed compound, although the ionic radii of the material was different. 

### 5.3. Decorated Structure

In these structures, the host material is decorated with nanoparticles of a secondary material. This method is employed to combine different types of materials, such as metals and carbon-based materials like graphene [18,19,20,21]. The existence of a semiconductor–semiconductor junction or a Schottky contact results in several sensing properties that alter the nature of heterostructures. Common methods used to decorate nanoparticles are sputtering, thermal evaporation, and solution. The second decorating material of the structure has to be applied in lower quantities to make it effective. 

**Figure 13:**
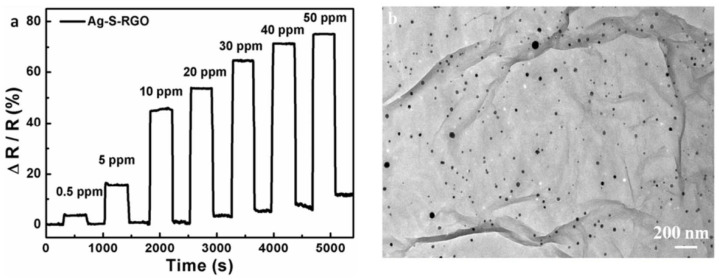
(**a**) Response of the Ag-S-RGO sensor as function of time (s) in various concentrations of NO_2_ gas and (**b**) TEM image of Ag nanoparticles on S-RGO sheets. Reprinted with permission from Ref. [119]. Copyright 2014, Huang, L. et al.

Gautam et al. found that the NH_3_ gas-sensing nature of graphene deposited using CVD showed enhanced sensitivity and improved recovery time after decorating with gold nanoparticles on the surface of graphene films [117]. Huang et al. prepared an NO_2_ sensor using sulfonated rGO decorated with Ag nanoparticles (as displayed in Figure 13). This device demonstrated quick responses to NO_2_ in comparison to other graphene-based sensors. When rGO decorated with Ag nanoparticles were put under 50 ppm of NO_2_, the sensor displayed a good sensitivity of 74.6% and a response and recovery time of 12 s and 20 s, respectively. Liu et al. reported on an H_2_ sensor using SWCNT decorated with Pt nanoparticles synthesized with the aerosol jet-printing technique [118]. After treating the SWCNT powder using acid to remove the catalyst and the carbonaceous impurities, the purified SWCNT was mixed into sodium dodecyl sulfate (SDS) and ethylene glycol (EG) solution. To the mixture, H_2_PtCl_6_·H_2_O was then added and reduced to form the Pt-SWCNT hybrid. Finally, printable ink was made from the hybrid nanoparticles. The ink was coated on an Si substrate to obtain a thin-film gas sensor. The sensor demonstrated high sensitivity to H_2_ with a limit of detection of 20 ppm. 

Nguyen et al. employed Co nanoparticles to modify the MWCNT synthesized on Al_2_O_3_ substrates, resulting in enhanced and highly promising NH_3_ gas sensitivity at room temperature [120]. The Co nanoparticles were distributed on the surface of the MWCNT film using the sputtering technique. Decorating with Co nanoparticles increased the thickness of the film to about 2 nm, and the roughness of the individual MWCNT surface was also increased. The sensor showed excellent reproducibility along with good selectivity to NH_3_ in comparison to alcohol and LPG. 

Wei et al. [121] worked on improving SWCNT sensing behavior towards NO_2_ by incorporating SnO_2_. SWCNTs with an average diameter ~0.8 nm were first synthesized using high-pressure CO disproportionation. Using the SWCNT-dispersed organometallic solution of Sn[OOCCH(C_2_H_5_)C4H9]_2,aq_. Tin (II) 2-ethylhexanoate ~90% in 2-ethylhexanoic acid, a SWCNT/SnO_2_ layer was formed with spin coating on an Al_2_O_3_ substrate with comb-designed Au electrodes. The hybrid layers were heated in air to eliminate the solvent with the help of an IR dryer at 150 °C for about 30 min, then further annealed at 500 °C for 35 min to form SnO_2_. In these structures, the SWCNT bundles were embedded into the SnO_2_ matrix. The SWCNTs/SnO_2_ hybrid sensor exhibited high sensitivity and fast recovery in comparison to the SnO_2_ sensor. The high sensitivity of the SWCNTs/SnO_2_ hybrid was due to the widening of the depletion layer at the grain boundaries of SnO_2_ and the interface of SWCNT/SnO_2_. 

Khalilian et al. used TiO_2_ nanoparticles decorated with MWCNT to produce a hybrid gas sensor for detecting O_2_ and methanol [122]. CVD was employed to obtain both the MWCNTs and the TiO_2_ nanoparticles under different conditions. The average diameter of the MWCNTs was 50 nm; the size of the TiO_2_ nanoparticles was ~5 nm. The surfaces of the MWCNTs were uniformly distributed with TiO_2_ nanoparticles. The *p*-*n* junction created at the interface between MWCNT and the TiO_2_ nanoparticles weakened the electron and hole pair recombination, thus enhancing the charge density when exposed to O_2_. It was found that the gas-sensor sensitivity depended on the sensitivity of the TiO_2_ nanoparticles. The optimized thickness of the TiO_2_ nanoparticle was 6 nm. When the layer of the TiO_2_ nanoparticle was too thick, it was found that the effect of the TiO_2_-MWCNTs heterostructure disappeared. 

Kaniyoor et al. compared the performances of a gas sensor based on graphene and MWCNTs decorated with Pt nanoparticle for detecting H_2_ at room temperature [123]. The hybrid sensors were synthesized using the drop-casting technique. The MWCNTs and graphene were prepared with the help of CVD and the thermal exfoliation technique. To decorate the nanocarbon, the H_2_PtCl_6_ solution was gradually mixed with graphene and MWCNT suspensions, and then the mixed solution containing NaOH and NaBH_4_ was reduced. The sensitivity of the Pt-graphene device was twice as great as the sensitivity of the Pt-MWCNT hybrid under low 4 vol% H_2_ concentration. 

Karaduman et al. compared the effects of Ag, Au, and Pt noble metallic nanoparticles on the rGO-sensing cahracteristics of NH_3_ at room temperature [123]. The nanoparticles were decorated using a chemical reduction technique. The Ag nanoparticle-based sensor had the best stability, sensitivity, response/recovery time, and selectivity. Modification with Ag nanoparticles resulted in strong adsorbtion of the NH_3_ molecules on the rGO nanosheet surface. Tang et al. also found that SWCNTs decorated with Pd noble metal nanoparticles improved the gas-sensing response to H_2_ [124]. 

**Figure 14:**
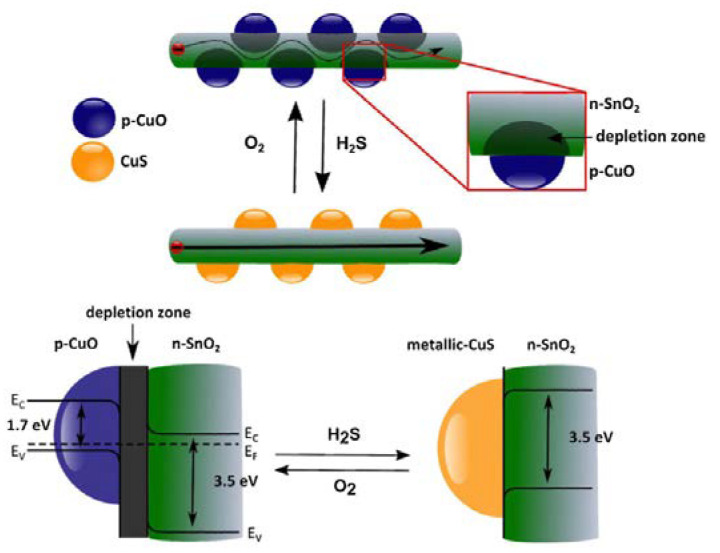
Schematic diagram of p-CuO particles decorated on the n-SnO_2_ surface, creating a pn junction. The effective conduction channel in the nanowire reduced due to depletion region, enhancing the resistance. Reprinted with permission from Ref. [125]. Copyright 2013, Shao, F. et al.

Shao et al. decorated n-SnO_2_ nanowires using p-CuO nanoparticles [125]. It was found that the catalytic activity of the p-type nanoclusters favored the detection of the specific analyte gas. The H_2_S-sensing performance was improved due to the decoration of CuO nanoparticles because of the strong chemical reaction of H_2_S and CuO, as seen in Figure 14. In that study, the CuO nanoparticles reacted with H_2_S, then sulfurized into CuS. Consequently, hydrogen, which was left out, was transferred to the host surface, serving as a reducing agent. This decreased the electrical resistance. 

Mashock et al. reported on the decoration of p-CuO nanowires with n-SnO_2_ nanoparticles. The depletion region was created between the p-CuO nanowires and the n-SnO_2_ nanoparticles, impinging the conducting core [126]. The electrical resistance increased after decorating with discrete SnO_2_ nanoparticles. Furthermore, they obtained a uniform coating, resulting in a core–shell assembly when the deposition time was doubled for the nanoparticles. It was found that the longer the deposition time, the higher the electrical resistance, producing a slight increase in sensitivity towards NH_3_ in comparison to the nanowires grown in a short timeframe. Two principles were proposed for the sensing nature: (1) transfering of electrons to the CuO nanowires from NH_3_ and lowering the concentration of the hole in the CuO nanowire, thereby enhancing the resistance, and (2) high concentration of electrons in SnO_2_ owing to the lower exposure of oxygen adsorbates, which makes the pn junction robust and blocks the transport of the hole close to the junction in the CuO nanowire, enhancing the nanowire resistance.

Jiao et al. reported on ZnO nanowire growth using the hydrothermal technique and decorated ZnO nanwires with PdO using sputtering [127]. The well-dispersed PdO along the ZnO nanowire surface delivered a higher surface area-to-volume ratio, increasing the area of gas adsorption. Studies found that decorating enhances the sensing response of p-type materials for gas-detecting applications [128,129]. Tan et al. used 1.5% of porous Fe_2_O_3_ decorated on NiO nanosheets. This increased the response to 170.9 and 107.9 toward the sensing of ethanol and methanol, respectively, at 255 °C, which is approximately 25 times greater than bare NiO nanosheets [130]. Decorating Fe_2_O_3_ on NiO also decreased the response time and recovery time. Cui et al. demonstrated that SnO_2_ nanocrystals decorated on MoS_2_ nanosheets resulted in higher sensitivity, good selectivity and stability, and reproducibility toward NO_2_ sensing in a dry environment; however, the MoS_2_ nanosheets alone were unstable under the same working environment without decorating with SnO_2_ nanocrystals [131].

R. Zhan et al. devoloped a strategy that enhanced the sensativity of the sensors by effectively confining Au nanoparticles with analyte molecules together in a short amount of time on a needle tip. They used the capillary force and Leidenfrost phenomenon and a small sensative region was created on a needle tip. The evaporated Au nanoparticles and analytes condensed on the needle tip, creating a small 0.5 nm area. Due to the high enrichment capability and reproducibility, surface-enhanced Raman scattering (SERS) was used to detect analytes with high sensitivity. It was obsered that with the SERS techinique, the limit of detection was lowered to 0.08 nM for crystal violet molecules, and for several other pesticides the detection limit was achieved in nM. They furthur applied this tehiniques with a mixture of pigments in a solution and achived high selectivity and sensitivity. Morever, they showed that the SERS techinique, with a highly thermal conductivity substrate, can open the possibility of universal applications wihout surface modification [132].

Zizzari et al. reported highly sensitive pressure sensors based on a thin membrane for calalytic reaction monitoring. In this research, elastic thin membranes were integrated with a reaction chamber to develop a highly sensitive membrane-based pressure sensor (MePS). The MePS was tested for different pressure, ranging from 2 and 50 Pa, which allowed dioxygen variation to be monitored in the order of μmol L^–1^ s^–1^ [133].

Wang et al. also reported highly sensitive pressure sensors based on electrospun membranes. A dielectric layer of an AgNW/TPU composite was used with the thermoplastic polyurethane elastomer rubber (TPU) electrospinning nanofiber membranes. The device was completed by printing the carbon nanotubes with flexible electrodes. The results showed a high sensitivity of 7.24 kPa^−1^ within the range of 9.0 × 10^−3^~0.98 kPa, and a low detection limit of 9.24 Pa was achieved. In addition to that, fast responsiveness of <55 ms was achieved in this research. Moreover, the authors reported remarkable stability, sensitivity, and durability of this flexible pressure sensor [134].

### 5.4. Core–Shell Structures

In core–shell structures, the host core material is completely covered by a secondary shell material, thereby increasing the interfacial area in spite of exposing the host material to ambient atmosphere. The host structure is usually a single crystal structure synthesized using PVD, hydrothermal, CVD, and other methods. The outer shell is polycrystalline and deposited by electrospinning, sputtering, spin coating, or ALD. These types of heterostructures have good potential for use in gas-sensing applications due to the wider combinations of various materials and surface interactions, namely, core–shell, shell–gas, shell–shell, and core–gas. It is important to look for compatible core–shell materials to enhance the heterojunction synergy. Furthermore, tuning shell–shell and shell–gas interactions is a good way to improve the sensing response, as the shell layer grain boundary can be decisive for the performance of sensors. For instance, the shell layer thickness can vary the sensor responses due to changes in the grain boundary characteristics. Woo et al. worked on the growth of a core n-ZnO nanowire coated with shell p-type Cr_2_O_3_ heterostructures to sense trimethylamine (TMA) [135]. That study showed that the Cr_2_O_3_ layer narrows the ZnO channel conduction, thereby enhancing the resistivity of the sensors in air, thus demonstrating strong deviations while interacting with the TMA-reducing gas. Moreover, Cr_2_O_3_ exhibited a strong catalytic nature toward methylamine decomposition. The p-type Cr_2_O_3_ layer was the dominant path for conduction in the core–shell structures.

Park et al. worked on improving the NO_2_-detecting characteristics of TeO_2_ nanorods by growing core–shell CuO/TeO_2_ nanorods [136]. TeO_2_, along with CuO, possesses p-type semiconducting properties. The sensing mechanism is based on the barrier-controlled carrier transport process. They reported on *pp* heterojunctions formed with TeO_2_ and CuO, and they found they effectively enhanced the sensing characteristics in comparison to the grain boundaries and nanorod–nanorod contact due to the higher interface area of the core–shell structure. Rivera et al. worked on sensing methanol using ZnO nanorods and coaxial core–shell ZnO/MgZnO structures [137]. That study found that a high concentration of oxygen vacancies in the core–shell structure led to improved gas-sensing charateristics in comparison to the ZnO nanorod. They also found that there was quick adsorption/desorption on the core–shell structure. They added the claim that the neutral oxygen vacancies’ energy level was shallow in comparison to the single charged oxygen vacancies’ energy level. Consequently, faster adsorption along with desorption processes occurred in the core-shell ZnO/MgZnO structures in comparison to the ZnO nanorods.

**Figure 15:**
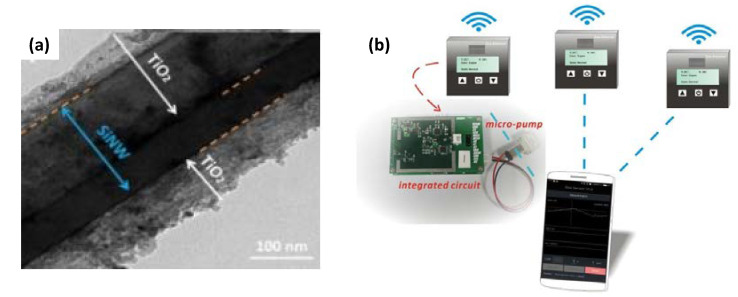
(**a**) TEM image of Si nanowires after being coated with TiO_2_. (**b**) The gas-sensor network linked by a mobile phone fitted with Andriod software, the integrated circuit, and the sensing trace depicted on cell phone figure are real pictures of products. Reprinted with permission from Ref. [138]. Copyright 2017, Liu, D. et al.

Liu et al. studied a Si nanowire/TiO_2_ array core–shell nanostructure synthesized using sol-gel and dropcasting methods, as depicted in Figure 15 [138]. The hybrid core–shell nanostructure exhibited excellent CH_4_ detection at room temperature. The sensor showed a linear characteristic response to CH_4_ in the 30–120 ppm range at a detection limit of 20 ppm. The enhanced performance was related to the depletion layer formed at the junction of the Si nanowires and TiO_2_. The oxygen adsorption, along with gas analyte adsorption, on the TiO_2_ surface induced variations in the thickness of the depletion layer; thus, the width of Si nanowire conductive channel cause a sensitive response to the gas analyte. This gas sensor constructed from Si nanowires/TiO_2_ operating at room temperature did not require an extra heating device, and it operates at the µW power level. This low power consumption is vital for sensing when used widely and connected to the Internet of Things (IoT). The innovation of these sensing materials at room temperature will enable the integration of gas sensors with wireless devices.

Li et al. fabricated SnO_2_, TiO_2_, and SnO_2_/TiO_2_ core–shell nanofibers using the electro-spinning technique [139]. The gas-sensing characteristics of these sensors were investigated to determine their selective response to gases, such as acetone, ethanol, formaldehyde, ammonia, methanol, and toluene. The core–shell nanofiber showed improved sensing reponse to acetone. This sensing operation depends on the carrier transport controlled by the barrier. Chen et al. also synthesized a core–shell structure consisting of TiO_2_ nanobelts and Sn_3_O_4_ nanosheets through a two-step hydrothermal process [27]. Arafat et al. studied various gas-sensing operations of an Al_2_O_3_/TiO_2_ core–shell nanostructure toward H_2_, CO, H_2_S, CH_3_OH, CH_4_, C_2_H_4_, C_2_H_5_OH, O_2_, and NO_2_ gases [140]. They found that the gas-sensing ability of a core–shell Al_2_O_3/_TiO_2_ depended on the electron tunneling-assisted surface depletion process. Al_2_O_3_ worked as an insulation layer on the TiO_2_ nanowire; however, electron tunneling occurred due to the small Al_2_O_3_ layer thickness where the electron could pass through the Al_2_O_3_ layer, forming a depletion layer in the core TiO_2_. Hence, the study found that the sensing mechanism worked by modulating the depletion region of TiO_2_.

### 5.5. Branched 1D Heterostructures

In the 1D branched morphology, the branch or brush-like heterostructure is synthesized by combining secondary nanowires on a host single nanowire. These exhibit interesting characteristics, similar to the decorated heterostructure, but with a significant increase in the surface area. In the branched heterostructure, the host offers the location for the growth of the secondary material. It is essential to control the uniformity, dimensions, crystallinity, and morphology of the host, as these factors have a direct impact on the growth of the secondary material. Generally, the techniques most often employed for preparaing 1D branched heterostructures are electrospinning and hydrothermal methods [47,141]. The synthesis of 1D branched nanostructures often requires a multi-step approach; however, it is usually possible to achieve the growth of highly crystalline secondary nanowires on a host nanowire [25,26].

**Figure 16:**
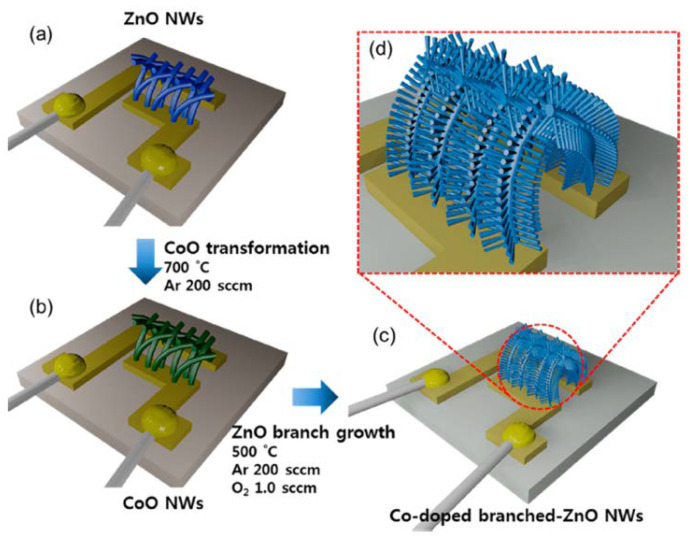
(**a**) ZnO nanowires, (**b**) transforming ZnO nanowires into CoO nanowires, (**c**,**d**) co-doped ZnO nanowire branches. Reprinted with permission from Ref. [142]. Copyright 2014, Woo, H.-S. et al.

Woo et al. deposited porous ZnO nanowires through Au-assisted VLS growth [142]. By a cation-exchange reaction, owing to the thermal evaporation of CoCl_2_ powder, the ZnO nanowires were changed into CoO nanowires. This resulted in the growth of co-deposited ZnO branches (Figure 16). In a p-xylene environment, there is great variation in resistance due to the presence of more free electrons, which results in improving the response of the gas sensor. This high response can also be related to the Co catalytic activity, enabling the dissociation of p-xylene, which is less reactive, into smaller reactive gases.

Kaur et al. reported on a NiO/ZnO branched heterostructure synthesized using the vapor transport condensation technique [25]. The angle maintained between the longer NiO nanowire and the shorter ZnO nanowire branches seemed fixed at about 50°, signifying the presence of lattice matching among both of the crystalline phases. Here, the selected area electron diffraction (SAED) analysis indicated the epitaxial growth of the ZnO nanowire (101) planes on the surface of strongly oriented NiO nanowire (200) crystallographic planes. In comparison to the host NiO nanowires, the NiO/ZnO branched heterostructure exhibited a higher sensing response to ethanol and acetone. The resistance increased after forming the heterostructure. Lou et al. also worked on the growth of TiO_2_-branched α-Fe_2_O_3_ nanorods synthesized using a two-step solution process for a trimethylamine gas sensor [47]. To tune the host to the guest mass ratio, the amount of secondary materials was adjusted, which affected the sensing properties of the heterostructures. The various studies reporting on gas sensor applications that were published in the past years are listed in Table 2.

## 6. Conclusions, Future Trends, and Challenges

This review presents an overview of the recent development of different materials and their heterostructures for chemical gas-sensor applications. We have shown the possibility of synthesizing various materials whose morphology and structures have unique sensing properties in relation to chemical compounds. In a significant number of the reviewed studies the research community focused on producing heterostructures using the hydrothermal method due to the ease of finding precursor materials and the simplicity of the hydrothermal and solvothermal methods for the synthesis. Electrospinning, ALD, PVD, and CVD methods are the other tehcniques that are widely explored for growing heterostructures, particularly core–shell, bilayer, decorated, and branched. These methods have advantages for growing selectively on patterned substrate, as well as preparing composites and multilayered structures. Combining various fabrication methods is also an effective strategy for obtaining different morphologies, heterostructures, and dimensions. Despite advancements in material science and nanotechnology, there are issues with these complex configurations and shortcomings that must be addressed. The decrease in nanostructure dimensions and trials make distinct interfaces degrade the long-term stability of these mateirals due to the coalescence of the nanoparticles and the diffusion along the interfaces. High temperatures are mostly required during the operation of gas sensors, which can lead to diffusion and the formation of mixed compounds, changing the electronic properties. However, heterostructures normally possess a lower optimal functioning temperature in comparison to their bare structures; hence, there can be partial mitigation of the diffusion process. Moreover, stability can be enhanced by employing highly crystalline 1D nanowires in place of nanoparticles, which suffer from diffusion and coalescence.

Reproducibility of the fabrication process and improvement in the performance of the sensors are needed before these devices can enter the market for mass scale production. It is important to employ techniques that give a high degree of control, such as ALD and sputtering processes. Consequently, characterization of the heterosructures is critical, and it needs to be performed carefully. The synergistic mechanisms that exist among heterostructure materials as well as their catalytic behaviors must also be considered. There is a strong analogy between conductometric gas sensing and the catalytic materials, which also needs to be carefully investigated. To fabricate high-performance sensing devices, the design of the materials is vital. Moreover, the active transducer needs an electrical connection to interface with the nearby electronics. These contact electrodes have a dramatic impact on the performances of these devices. The choice of contact materials and the deposition techniques varies the sensing properties.

There are also challenges that persist when integrating nanostructured heterojunctions with silicon-based devices, as technology is advancing with IoT and smart devices. Commercial sensors need to perform diagnostics and communicate and interface with the network configuration. Thus, the stages needed for fabricating heterojunctions are not compatible with Si processing and the high temperatures required for chemical sensors. In this case, heterostructures can be directly deposited using ink-jet printing or hotplates. These growth techniques require careful attention to processing methods that employ temperatures that match the substrates and do not use a destructive chemical precursor.

Composite nanoheterostructure devices can revolutionize gas sensing, but more discoveries and experiments are need to recognize the full potential of these materials. This review concentrated on the configurations and materials that could be used to prepare heterojunctions to show their advantages and properties. The composite materials obtained by combining metal oxides with rGO and other carbon-based materials, transition metal dichalcogenides, and phosphorene have proven to be important for gas-sensing applications. Therefore, the final goal of this review was to summarize the state of the art and to stimulate curiosity among readers to inspire them to further investigate these exceptional classes of nanomaterials.

## Figures and Tables

**Table 1:** Comparison of various types of gas sensors. Reprinted with permission from Ref. [37]. Copyright 2007, Korotcenkov, G.

Parameters	Types of Gas Sensors				
	SMO Gas Sensors	Catalytic Combustion Gas Sensors	Electro Chemical Gas Sensors	Thermal Conductivity Gas Sensors	Infrared Absorption Gas Sensors
Sensitivity	E	G	G	P	E
Accuracy	G	G	G	G	E
Selectivity	F	P	G	P	E
Response time	E	G	F	G	F
Stability	G	G	P	G	G
Durability	G	G	F	G	E
Maintenance	E	E	G	G	F
Cost	E	E	G	G	F
Suitability to portable instruments	E	G	F	G	P

E: excellent, G: good, F: fair, P: poor.

**Table 2:** Works reported and published in the past years for gas-sensor applications.

Composition	Synthesis Route	Analyte Gas	Performance	Reference
PANI thin film	Chemical oxidative polymerization process	NO_2_	12.10% at 100 ppm T_res_ = 11 s T_rec_ = 7 min	[77]
Ag-doped PPy thin film	In-situ oxidation	NO_2_	68% at 100 ppm NO_2_ T_res_ = 148 s T_rec_ = 500 s	[80]
Single SWCNT	CVD	NO_2_	1000 fold @ 200 ppm T_res_ = 2–10 s T_rec_ = 12 h	[92]
MWCNT	CVD	N_2_	T_res_ = 10 s T_rec_ = 10 s	[103]
Sulphonated rGO decorated with Ag nanoparticles	Wet chemical pathway	NO_2_	74.6% @ 50 ppm T_res_ = 12 s T_rec_ = 20 s	[119]
Multilayered graphene	CVD	NO_2_	6% @ 1 ppm T_res_ = 1800 s	[89]
SnO_2_/CuO multilayered film	PLD	H_2_S	2.7 × 10^4^ @ 20 ppm T_res_ = 2 s	[50]
SnO_2_/TiO_2_ multilayered film	Sputtering	H_2_S	1.6 × 10^4^ @ 10 ppm T_res_ = 3.2 s T_rec_ = 2.4 s	[107]
WO_3_/SnO_2_ mixed compund	Sol-precipitation	NO_2_	186 @ 200 ppm	[116]
VO_2_–SWCNT mixed compound	Hydrothermal flow synthesis	NH_3_	10% @ 45 ppm T_res_ = 7 s T_rec_ = 30 min	[110]
PANI-CNT mixed compound	Interfacial polymerization	NH_3_	418% @ 4 ppm T_res_ = 18 s T_rec_ = 46 s	[113]
SWCNT decorated with Pt nanoparticles	Aerosol jet printing	H_2_	4% @ 200 ppm	[118]
MWCNTs with TiO_2_ nanoparticles	CVD	O_2_	15% @ 1000 ppm T_res_ = 50 s T_rec_ = 100 s	[122]
Fe_2_O_3_ decorated on NiO nanosheets	Thermal decomposition nanosheets	Ethanol	170 @ 100 ppm T_res_ = 0.1 s T_rec_ = 11.4 s	[130]
ZnO nanwires decorated with PdO	Hydrothermal and sputtering	H_2_	3.6 @ 500 ppm T_res_ = 207 s T_rec_ = 92 s	[127]
Coreshell CuO@TeO_2_ nanorods	Thermal oxidation and sputtering	NO_2_	424.91 @ 10 ppm	[136]
SnO_2_@TiO_2_ core–shell nanofibers	Electro-spinning method	Acetone	13.5 @ 100 ppm T_res_ = 2 s T_rec_ = 60 s	[139]
NiO/ZnO-branched heterostructures	Vapor phase growth technique	Ethanol	6.7 @ 50 ppm	[25]

## Data Availability

Not applicable.
